# On the origin of glioma

**DOI:** 10.3109/03009734.2012.658976

**Published:** 2012-04-19

**Authors:** Yiwen Jiang, Lene Uhrbom

**Affiliations:** Uppsala University, Department of Immunology, Genetics and Pathology, Rudbeck Laboratory, SE-75185 Uppsala, Sweden

**Keywords:** Astrocyte, cell of origin, glioma, glioma-initiating cell, neural stem cell, oligodendrocyte precursor cell

## Abstract

Glioma is the most frequent primary brain tumor of adults that has a presumably glial origin. Although our knowledge regarding molecular mechanisms and signaling pathways involved in gliomagenesis has increased immensely during the past decade, high-grade glioma remains a lethal disease with dismal prognosis. The failure of current therapies has to a large extent been ascribed the functional heterogeneity of glioma cells. One reason for this heterogeneity is most certainly the large number of variations in genetic alterations that can be found in high-grade gliomas. Another factor that may influence glioma heterogeneity could be the cell type from which the glioma is initiated. The cell of origin for glioma is still undefined, and additional knowledge about this issue may prove critical for a more complete understanding of glioma biology. Based on information from patients, developmental biology, and experimental glioma models, the most putative target cells include astrocytes, neural stem cells, and oligodendrocyte precursor cells, which are all discussed in more detail in this article. Animal modeling of glioma suggests that these three cell types have the capability to be the origin of glioma, and we have reason to believe that, depending on the initiating cell type, prognosis and response to therapy may be significantly different. Thus, it is essential to explore further the role of cellular origin in glioma.

## Histopathological and molecular classification of glioma

Glioma is the most common primary intracranial tumor with a worldwide incidence of approximately 7 out of 100,000 individuals per year. Despite extensive efforts and huge progress in our knowledge about the underlying mechanisms of glioma development during the last decades, there is still no cure, and the median survival for patients diagnosed with the most frequent and malignant form of glioma, glioblastoma (GBM), remains at 12–15 months (1).

Gliomas are graded from I to IV according to the World Health Organization (WHO) malignancy scale. Grade I lesions are benign and relatively circumscribed with a slow proliferation rate and include the most common glioma of children, pilocytic astrocytoma. Grade II tumors, including astrocytoma, oligodendroglioma, and oligoastrocytoma, also have a slow growth rate and a high degree of cellular differentiation, but unlike pilocytic astrocytomas these tumors grow diffusely into the normal brain parenchyma and are prone to malignant progression. Grade III tumors include anaplastic astrocytoma, anaplastic oligodendroglioma, and anaplastic oligoastrocytoma, which are characterized by a higher cellular density and the ample existence of atypia and mitotic cells. Grade IV tumors are the most malignant and also the most frequent gliomas and include glioblastoma and gliosarcoma. These tumors display, in addition to grade III features, either or both of the hallmarks of grade IV glioma: microvascular proliferations and pseudopalisading necrosis.

Large-scale genomic analyses of human glioblastoma have identified numerous mutated genes, and the data propose that three core signaling pathways are altered in the majority of the tumors: the receptor tyrosine kinase (RTK)/RAS/PI3K pathway, where *EGFR* amplification, *PDGFRA* overexpression or amplification, and *PTEN* inactivation are among the most common mutations; the p53 pathway, where inactivation of p53 and p14^Arf^ are key events; and the RB pathway, with inactivation of RB and p16^Ink4a^ as the most frequent alterations (1,2). In addition, when the large-scale genomics data were combined with large-scale gene expression and proteome analyses of glioblastomas, that by mere histopathology were inseparable, a classification based on the tumors' molecular profiles into 3–4 subtypes could be generated (3-5).

High-grade gliomas display a large degree of heterogeneity, both among tumors with the same diagnosis and among tumor cells within the same tumor. This morphological, cellular, and molecular heterogeneity is probably to a large extent caused by variations in the combinations of genetic and epigenetic mutations that have occurred during glioma development. As mentioned above, we now have substantial knowledge regarding this aspect of glioma. An additional contributor to glioma heterogeneity could be the cell type from which the glioma originates, which is still undefined. This aspect has been investigated using various mouse models of glioma (Table I), but transfer of that knowledge to human glioma has not been convincingly done. Recent findings that committed glial cells have the potential to be the cell of origin for glioma (6-8) have renewed the attention on the glioma cell of origin. We believe that to truly understand the complex biology of gliomas it is essential to know both the genetic alterations of that tumor and from which cell type it has arisen, information that could build the foundation for designing new, more efficient, and more specific treatment strategies. Below we will discuss the most studied candidate cells of origin for glioma, namely the astrocyte, the neural stem cell (NSC), and the oligodendrocyte precursor cell (OPC).

**Table I.** Mouse models with confirmed or candidate cell of origin for glioma.

Cell of origin	Promoter	Deleted or inactivated genes	Overexpressed or activated genes	Ref
Astrocyte	NA	p16^Ink4a^ and p19^Arf^	Mutant EGFR	(29)
	GFAP		v-Src	(30)
	GFAP		Ha-Ras	(31)
	GFAP	pRb, p107, p130		(32)
	GFAP (RCAS-tv-a)		PDGF-B	(21)
Neural stem or progenitor cell	Nestin-CreERT2	Nf1, p53, and/or Pten		(63)
	GFAP-Cre		H-Ras and Akt	(24)
	Nestin (RCAS-tv-a)		K-Ras and Akt	(64)
	NA	p16^Ink4a^ and p19^Arf^	Mutant EGFR	(29)
Oligodendrocyte precursor cell	NA	p53	Ras	(74)
	CNP (RCAS-tv-a)		PDGF-B	(8)
	S100β		Mutant EGFR	(6)
	GFAP-Cre or Nestin-Cre	Nf1 and p53		(7)

NA = not applicable.

## Relationship between cell of origin, cell of mutation, and glioma-derived cancer stem cells

There are histopathological similarities between gliomas and normal glial cells of the brain, as evidenced by immunostainings showing glial tumors to express numerous glial markers. This was the basis for the assumption made almost a century ago that gliomas would originate from a glial cell type. Many years later, with the discovery of glial stem and progenitor cells (9-12), the interest was further narrowed to immature glial cells. The fairly recent identification of glioma-derived cancer stem cells or glioma-initiating cells (GICs) in high-grade gliomas (13–15) was interpreted as a further support for an immature cell as the origin of glioma, since GICs were shown to exhibit many of the same properties as NSCs such as expression of NSC markers, the ability of extended self-renewal, and the capacity to differentiate into cells expressing glial and neuronal markers. These cells are thought to sustain tumor development, and they have been shown to resist radiotherapy (16) and chemotherapy (17), making them a vital target for drug development. However, when during tumor development, and how, GICs occur is still unknown. Very recently an additional complexity to this matter was discovered when it was shown in an experimental model of glioma that the cell of mutation and the cell of origin could be different cell types (7). When *p53* and *NF1* were homozygously mutated in NSCs using the MADM (mosaic analysis with double markers) mouse model, malignant transformation generating gliomas occurred only in OPCs. Whether this means that OPCs in general are more susceptible to oncogenic transformation than other glial cell types, or if OPCs are more susceptible to this particular combination of genetic alterations is yet to be determined.

## Mouse models to study origin of glioma

At the time of diagnosis the majority of glioma patients are already in a late stage of the disease, which makes it unfeasible to study the cell of origin in humans. To address this issue, results from genetically modified mouse models can be used (Table I). In many (but not all) instances these models were initially made with the intention to study the causal effects of specific genetic mutations commonly found in human glioma. To establish models that would be relevant to human glioma various neural and glial promoters have been utilized to direct the genetic lesions to glial cells, which has generated information also about the putative cellular origin of these tumors. The models can be divided into two categories: germline modification models and somatic cell gene transfer models.

In germline modification models gliomas are generated by depletion of tumor suppressor genes or overexpression of oncogenes, traits that are inherited in a Mendelian pattern. By using cell type-specific promoters, genetic modifications can be directed to specific neural and glial cell lineages. The application of the *Cre/loxP* technology further allows the genetic alterations to happen at a specific time in development and/or in a specified region or cell type (18). Recently, as already mentioned, the MADM-based model was used to study glioma development, which allowed lineage tracing to follow the earliest events of tumor development and identify the premalignant cell of mutation and the malignant cell of origin (7).

In somatic cell gene transfer models the genetic alterations are delivered to specific somatic cell types by retroviral infection. Stable integration of the retroviral genome into the host cell genome followed by expression of virally transduced genes can cause cell transformation and tumor development. To obtain cell type specific retroviral infection the RCAS (replication competent ALV splice acceptor)/tv-a system has been extensively used to study glioma development (19). RCAS is derived from the avian sarcoma-leukosis virus family, and its receptor, tv-a, is not present in the mammalian genome. RCAS virus can be manipulated to express a gene of choice, propagated in cultured chicken cells, and injected into tv-a transgenic mice where it will infect dividing cells. Several mouse lines have been generated that express tv-a under the control of glial-specific promoters (8,20,21), which allows for targeted infection by RCAS through stereotactic injections to a defined location of the brain and at a specified time in development (22). The tv-a mouse lines have been crossed with mice deficient for tumor suppressor genes, e.g. *Ink4a*, *Arf*, or combined loss of both (*Cdkn2a*), to investigate how combinations of mutations affect glioma development (23). Also a lentivirus-based glioma model has been developed recently where lentiviral vectors containing activated oncogenes flanked by *loxP* sites were injected into GFAP-*Cre* transgenic mouse brains (24).

## Astrocytes as putative cell of origin for glioma

For a long time, before the discovery of adult NSCs, astrocytes were thought to be the only dividing cells in the adult brain (25). That, together with the fact that the astrocytic marker GFAP could be frequently found in human glioma tissue, made astrocytes a valid candidate to be the cell of origin for glioma (26). One concern with this hypothesis has been that it presumes that dedifferentiation should take place to give rise to the seemingly more immature cells present in high-grade glioma. Evidence that astrocytes could hold the capacity to dedifferentiate has been produced by several labs. Long-time exposure of mature astrocytes to TGF converted the cells first to radial glial cells and later to neural stem cell-like cells (27). When these dedifferentiated astrocytes were exposed to gamma radiation, they became immortalized and could induce high-grade gliomas after brain engrafting (28). Further, primary astrocytes obtained from newborn *Ink4a/Arf* ^-/-^ mice that were cultured in serum-free media supplemented with EGF underwent a rapid change in morphology and after 10 days started to form neurospheres that demonstrated a complete loss of GFAP but expressed nestin and A2B5 (29). These cells were shown to be equally as sensitive as NSCs to transformation with a mutated epidermal growth factor receptor (EGFR), and orthotopic transplantation of transduced cells to mice caused development of high-grade gliomas.

To analyze the potential of astrocytes to act as cell of origin in mouse glioma models, the GFAP promoter has been frequently used to control expression of many different oncogenic mutations. Some examples are: 1) Expression of a constitutively activated tyrosine kinase *v-src* in GFAP-expressing cells was shown to induce astrocytomas with a less than 20% penetrance (30). 2) Expression of an activated form of *H-Ras* in astrocytes led to astrocytoma formation in over 95% of the mice, and there was a dose-dependent correlation between Ras expression and tumor grade (31). 3) Inactivation of pRb, p107, and p130 by a truncated SV40 T-antigen (*T121*) in GFAP-expressing cells induced astrocytomas in 100% of the mice, and the process was accelerated by heterozygous loss of *PTEN* (32). 4) Somatic delivery of *PDGF-B* to astrocytes of newborn mice using the RCAS/tv-a system resulted in oligodendrogliomas or mixed oligoastrocytomas in about 40% of the mice by the age of 12 weeks (21). In all these studies the GFAP promoter was used, and astrocytes were the assumed target cells. However, there is a possibility that the tumors could have originated from other cell types since GFAP is also expressed by radial glial cells (33) and NSCs of the adult subventricular zone (34).

## The potential of the NSC as origin for glioma

The NSC of the subventricular zone (SVZ) has been a candidate cell of origin for glioma for many decades. Early observations from patients showed that human brain tumors were frequently located near the SVZ, suggesting that they had originated from the ‘subependymal plate’ (35), a notion that was recently corroborated in a large series of patients which showed that 93% of cases contacted the lateral ventricular wall (36). A series of studies performed in the 1960s and 1970s showed that an area adjacent to the cerebral ventricles was more sensitive than the rest of the brain to chemical and virus-induced gliomagenesis (37,38). This was the same region that in rodents and primates had been found to contain cells with mitotic activity regarded as NSCs (39,40). The true identification of a bona fide adult NSC was first made in the mouse in the 1990s, followed by similar findings in other species, such as monkey and human (9-11,41). These findings established that adult NSCs were mainly located in the SVZ of the lateral ventricles and in the dentate gyrus of the hippocampus. These NSCs have been shown to express several cell surface markers such as nestin (10), Sox2 (42), CD133 (43), and GFAP including GFAP delta (9,44), but none of these markers is unique to NSCs.

Another indication that NSCs could be the cell of origin for glioma is that signaling pathways, which are important for regulating self-renewal, proliferation, and differentiation of NSCs, are commonly altered in gliomas. The role of epidermal growth factor receptor (EGFR) signaling has been widely studied in both NSCs and glioma development. During rat brain development, EGF expression can be detected as early as on embryonic day 14 and remains detectable in the adult brain-stem, cerebellum, hippocampus, diencephalon, and telencephalon (45). EGFR signaling can induce proliferation of a small cluster of cells isolated from the adult mouse striatum, and these cells exhibit properties of NSCs (10). EGFR signaling can also regulate the fate decision of neural stem/progenitor cells and thus contribute to adult neurogenesis (46). The *EGFR* gene is mutated or amplified in 45% of glioblastomas (2). The most common mutation is EGFRvIII, which has a deletion of exons 2–7 generating a ligand-independent, constitutively activated extracellular domain (47-49). Another example is PTEN, a protein tyrosine phosphatase that negatively regulates the AKT/PKB signaling pathway. PTEN can suppress proliferation of NSCs, and mice deficient for PTEN have enlarged and abnormally structured brains (50). PTEN is mutated or homozygously deleted in 36% of glioblastomas (2). BMI-1, a member of the polycomb group of transcriptional repressors regulating expression of the *CDKN2a* locus, is a third example. BMI-l depletion in mice results in postnatal growth retardation and neurological defects (51) due to reduced self-renewal capacity of BMI-1-deficient NSCs (52). BMI-1 copy number variations have been observed in a subset of gliomas (53), and targeting BMI-1 by microRNA-128 could inhibit glioma proliferation (54). A fourth example is secreted signals, such as Notch and Wnt, which have been extensively studied in normal neural development. Notch acts as a key regulator of plasticity in the brain, balancing the number of NSCs and their progenies to maintain the stem cell niche, by regulating the cell cycle (55,56). Notch-1 and its ligands, Delta-like-1 and Jagged-1, have been shown to be overexpressed by many glioma cell lines (57). Moreover, merely interfering with either Notch-1 or its ligands can induce apoptosis and inhibit proliferation in a number of glioma cell lines. Wnt is necessary for the development of the midbrain and rostral metencephalon in the mouse (58). A recent study showed that Wnt signaling was absent when neural stem cells were dividing asymmetrically and was up-regulated upon symmetrical division. Thus, Wnt signaling could regulate the size of the stem cell pool (59). Although aberrant Wnt signaling is more common in a subset of medulloblastomas, and mutations of Wnt pathway genes are rare in gliomas, a close correlation of the Wnt/-catenin pathway with glioma progression has recently been found (60).

A third line of support for the NSC as a potential cellular origin for glioma comes from mouse models. Mice carrying combined germline mutation of *p53* and conditional knock-out of *Nf1* developed a spectrum of astrocytomas with 100% penetrance (61). Histopathology analysis of these tumors demonstrated that presymptomatic lesions were located within the SVZ, suggesting that SVZ stem cells may serve as cells of origin. Additional mutations of one or both alleles of *Pten* enabled tumor progression to anaplastic astrocytomas or glioblastoma (62). In this model, ectopic migration of *Nf1;p53;Pten-*deficient neural stem/progenitor lineage cells could be observed. Further studies from the same group proved that the inactivation of *p53*, *Nf1*, or *Pten* in adult neural stem/progenitor cells was sufficient to induce astrocytoma formation (63). In contrast, the same combination of mutations in non-neurogenic regions of adult mice did not result in tumor formation. A similar study whereby H-RAS and AKT were activated in GFAP + cells of the hippocampus, the SVZ or the cortex of adult mice heterozygous for *p53* showed that glioblastomas could only robustly develop when transformation happened in the first two areas (24). This observation was consistent with previous studies performed in newborn mice where Nestin + neural stem/progenitor cells were shown to be more sensitive to K-RAS and AKT transformation than GFAP + astrocytes (64). However, as already discussed, nestin and GFAP promoters are not unique to NSCs, since Nestin is expressed also by glial progenitor cells and astrocytes, and GFAP is additionally expressed by radial glial cells and astrocytes.

An alternative *in vivo* strategy to determine the potential of a cell type to be the cellular origin of glioma is to manipulate cells *in vitro* followed by transplantation to mice. NSC cultures have been established from *Ink4a/Arf* ^-/-^ mice that were transduced with retrovirus encoding a mutated form of the EGFR. These cells could induce high-grade gliomas when orthotopically transplanted into the brains of *SCID* mice (29). Another study using human NSC cultures established from glioma samples demonstrated that after 30 passages, when most cells had entered senescence, a spontaneous immortalized NSC clone emerged. This clone exhibited the characteristics of GICs, such as extensive self-renewal and high *in vivo* tumorigenicity (65). However, it should be noted that *in vitro* culture conditions are very different from the *in vivo* microenvironment and may cause a clonal selection of non-representative cells.

## Strong experimental support for the OPC as cell of origin for glioma

The OPC, marked by expression of e.g. NG2 (66), PDGFR (67), A2B5 (68), and CNPase (69), is the major dividing cell population in the adult brain and gives rise to oligodendrocytes. The broad distribution of OPCs in the SVZ, white matter, and gray matter, together with their proliferative ability, makes them a susceptible target to oncogenic transformation. In support of this, the PDGFRα signaling pathway involved in normal development of oligodendrocytes by controlling proliferation and migration of OPCs (70) is also commonly altered in gliomas (1,2). Also markers connected with OPCs, such as NG2 and PDGFR, have been found readily expressed in oligodendrogliomas, pilocytic astrocytomas, and glioblastomas (71). Further, integrated genomic analysis revealed that alteration of PDGFR was a key feature of the proneural subtype of glioblastomas (4).

OPCs were, for many years, regarded as irreversibly committed precursor cells that could only give rise to oligodendrocytes *in vivo* and oligodendrocytes and type-2 astrocytes in culture (72). Now we know that OPCs are more plastic and can be converted to immature multipotent cells *in vitro* that in turn can give rise to neurons, type-1 astrocytes, and oligodendrocytes (73). Further, it has been shown that OPCs can be transformed to GIC-like cells by a combination of Ras activation and p53 depletion, and these cells were highly efficient in inducing secondary tumors (74). Interestingly, the gene expression profile of the transformed OPCs showed that these cells had undergone global reprogramming and were more similar to NSCs than OPCs.

More solid evidence pointing to OPCs as cell of origin in glioma has emerged in recent years. In our laboratory we had observed that the majority of gliomas induced in *Ntv-a* and *Gtv-a* mice had expression of NG2 in their tumors. This was true for both combined AKT + KRAS (75) and PDGF-B (23) induced tumors, and, in addition, tumor cells had often lost their expression of nestin or GFAP. We reasoned that the oncogenic signaling somehow could drive the tumor cells into an ‘optimal state of differentiation’ for tumor development to occur, and based on the NG2 positivity that state was putatively an OPC. To analyze if OPCs could serve as cell of origin for glioma we developed a new tv-a transgenic mouse line, *Ctv-a*, in which viral infection could be targeted to OPCs expressing 2', 3'-cyclic nucleotide 3'-phosphodiesterase (CNP). CNP is a highly specific marker that in the central nervous system is only expressed late in OPC development and in mature oligodendrocytes. In the *Ctv-a* mouse we could induce low-grade oligodendroglioma with RCAS-PDGF-B. Although the cells that were infected were CNP+, the tumor cells were always CNP- but expressed other earlier OPC markers such as SOX2, SOX10, OLIG2, NG2, and PDGFR, which we interpreted as a slight dedifferentiation of tumor cells (8). Subsequently, corroborating our finding that OPCs could serve as cell of origin, gliomas generated in the S100β-vErb transgenic mouse model were deduced to have an OPC origin based on developmental and gene expression studies (6). In a very recent study using the MADM-based lineage tracing model to mutate sporadically *p53/Nf1* in NSCs, aberrant growth of pre-tumor lesions could only be found in cells that had differentiated along the oligodendrocyte lineage to become OPCs, and not in any of the other neural cell lineages or in the NSCs themselves (7). As a proof of concept, OPCs could also be directly transformed by *p53/Nf1* mutations *in vitro*, and the induced gliomas were indistinguishable from those initiated in NSCs. This is the first study to show that the cell of mutation and the cell of origin could be distinct cells, and further support the potential of OPCs to be the cell of origin for glioma. Due to the lack of unique markers for NSCs and astrocytes we argue that until now the most solid data from mouse modeling come from the OPC studies. The CNP promoter is highly specific to OPCs, and many of the other markers (e.g. NG2 and PDGFR) that were used to confirm the OPC origin in these studies (6–8) also have a narrow and rather specific expression pattern.

## Conclusion and perspective

**Figure 1.**
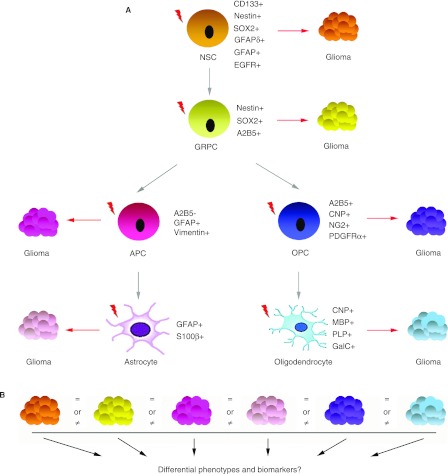
Putative glioma cell of origin and its role for glioma development. A: A simplified schematic representation of glial cell development from the multipotent CNS stem cell into astrocytes and oligodendrocytes, where each stage is defined by the expression of markers. Red lightings indicate the occurrence of an oncogenic event that will induce glioma development. B: The role of cellular origin for glioma initiation, progression, recurrence and response to therapy remains largely unknown. The search for biomarkers that could distinguish phenotypically distinct glioma subgroups based on cell of origin could be one path towards elucidating this matter. NSC = neural stem cell; GRPC = glial restricted progenitor cell; APC = astrocyte precursor cell; OPC = oligodendrocyte precursor cell.

The cell of origin for glioma remains unresolved, but experimental *in vivo* data show that many cell types of the brain may be capable of being that cell (Figure 1A). This suggests that human glioma can develop from many different cell types, and if adding to that the large collection of genetic alterations that have been identified in these tumors the complexity of glioma biology becomes apparent. We think that it could prove essential to take into account the dimension of cellular origin, since most likely this will give a more accurate representation of the properties of a particular glioma and thereby a better prediction of how the patient will respond to different therapeutic strategies. Therefore, it is important to continue to study the cell of origin for glioma, to investigate how the originating cell type affects glioma initiation, progression, recurrence, and response to therapy, and to identify biomarkers differentiating cellular origin that could be of predictive and therapeutic importance (Figure 1B).

Several strategies could be applied to explore cellular origin. Mouse models are powerful tools to study human disease. More cell type specific promoters are needed to be able to drive the initial oncogenic event within a well-defined cell population along the glial development axis. Optimal promoter candidates should have a narrow expression window during normal glial development. E.g. by combining the MADM-based lineage tracing method with other promoters and additional genetic mutations, consequences of novel combinations of genetic events and cell of origin could be analyzed *in vivo*.

Another path could be to use cross-species genomics. This is a strategy that has been successfully used to identify the cell of origin for specific subtypes of human medulloblastoma and ependymoma (76). In these studies the global gene expression profiles of defined populations of normal mouse cells, from different stages of development and specified locations of the brain, were compared to the profiles of different subtypes of human brain tumors. We suggest using this method in a slightly different way to compare gene expression profiles of mouse GICs derived from different and specified cellular origin to a large set of human GICs, with the aim to identify subclasses of human gliomas based on cellular origin. Our group has established GICs from experimentally induced mouse glioblastoma-like tumors with defined cellular origin, and we have found intriguing phenotypic differences of the GICs despite the fact that they have all been derived from grade IV tumors and are driven by the same initiating event (Y.J. and L.U., unpublished data). Global gene expression analysis is underway with the aim to stratify patient-derived GICs and to identify predicative and therapeutic biomarkers that are greatly needed for this incurable disease.
